# 免疫组化法检测非小细胞肺癌*EGFR*突变的进展

**DOI:** 10.3779/j.issn.1009-3419.2014.09.11

**Published:** 2014-09-20

**Authors:** 畅 刘, 东波 徐, 殿胜 钟

**Affiliations:** 1 300052 天津，天津医科大学总医院肿瘤内科 Department of Medical Oncology, Tianjin Medical University General Hospital, Tianjin 300052, China; 2 天津医科大学病理学教研室 Department of Pathology, Tianjin Medical University, Tianjin 300070, China

**Keywords:** 肺肿瘤, 表皮生长因子受体, 突变, 免疫组化法, Lung neoplasms, Epidermal growth factor receptor, Mutation, Immunohistochemistry

## Abstract

近些年来，人们越来越认识到，存在表皮生长因子受体（epidermal growth factor receptor, EGFR）突变的非小细胞肺癌（non-small cell lung cancer, NSCLC）患者对靶向药物EGFR酪氨酸激酶抑制剂（EGFR-tyrosine kinase inhibitor, EGFR-TKI）的治疗有良好反应。目前，检测*EGFR*突变应用最多且较为可靠的是以DNA分子为基础的检测（如DNA测序）方法，但此法操作繁琐，耗时长，花费高，对样本要求严格。相比之下，免疫组织化学法（immunohistochemistry, IHC）则充分弥补了上述缺陷，可作为*EGFR*突变筛查的辅助手段。但影响其结果的因素较多，如不同的免疫组化染色方法、不同抗原修复液的选择及不同的结果评判标准等，因此此法尚未广泛应用于临床，仅处于研究阶段。本文通过检索不同研究者应用免疫组化法对NSCLC患者进行EGFR突变检测的相关文献，进一步讨论如何合理应用免疫组化法检测EGFR突变可发挥其临床应用的最大价值。

## Abstract (en)

In recent years, it has been well known that non-small cell lung cancer (NSCLC) patients with mutations of epidermal growth factor receptor (EGFR) response beter to EGFR-tyrosine kinase inhibitor treatment. Although DNA-based assays (*e.g*. DNA sequencing) are the most frequently used and a relatively reliable method to detect *EGFR* mutations, they are complex, time-consuming and relatively expensive for routine use in clinical laboratories, besides they require high quality tumor samples. In contrast, the immunohistochemistry (IHC) methods make up fully for the above shortcomings and can serve as screening tests for *EGFR* mutations. However, there are many factors that can influence the results of IHC methods, such as different staining procedures, different antigen retrieval solutions and di?erent sets of criteria, *etc*. Tus the IHC methods for detecting *EGFR* mutations have not been widely used in clinic and only in the research stage. Tis article reviews the use of IHC methods by different researchers and further discusses how to make the IHC methods work best for the detection of *EGFR* mutations.

目前，全世界范围内肺癌的发病率和病死率居各类恶性肿瘤之首^[1]^。大部分肺癌患者诊断时已是晚期而丧失了手术机会，也因此无法获得手术标本^[2]^。约70%的肺癌患者是靠小的活检标本和/或细胞学切片标本诊断的，并且这些小标本是仅有的可用于突变检测的材料^[3, 4]^。经典的DNA直接测序法可检测所有已知和未知突变，被称为表皮生长因子受体(epidermal growth factor receptor, *EGFR*)突变检测的"金标准"，但其灵敏度较低，对样本所含肿瘤细胞数量要求较高，仅能对含量大于30%的突变基因进行检测^[5]^，当其用于小标本检测时，会使其假阴性率大幅增加^[4, 5]^。因此，相当一部分本可以从EGFR酪氨酸激酶抑制剂(EGFR-tyrosine kinase inhibitor, EGFR-TKI)获益的患者仍不能被检出。随着介入影像学和微创活检技术的进展，如支气管内超声引导下经支气管针吸活检术(endobronchial ultrasound-guided transbronchial needle aspiration, EBUS-TBNA)，应用小标本进行临床病理检测的趋势势必会延续^[6]^。近些年来随着分子水平技术的发展，涌现出一些检测基因突变更为灵敏的方法，如应用特异性探针的实时定量聚合酶链式反应(TaqMan PCR assay)、扩增阻滞突变系统技术(amplified refractory mutation system, A R MS)、聚合酶链式反应-单链构象多态性分析(PCR-single-strand conformation polymorphism, PCR-SSCP)、变性高效液相色谱分析(denaturing highperformance liquid chromatography, dHPLC)和高分辨率熔解曲线分析(high-resolution melting analysis, HRMA)等^[7-10]^。但这些方法或因其价格昂贵，操作复杂，耗时长，对实验环境、操作人员水平及设备要求高，尚未广泛应用于临床。免疫组织化学染色法较分子水平的检测手段价格低廉，操作简便、迅速，可在几乎所有病理实验室开展。因此，对突变特异性抗体的免疫组化法应用可作为分子水平检测方法的一种辅助手段。

已有大量文献及临床资料^[11, 12]^证实存在EGFR基因突变的非小细胞肺癌(non-small cell lung cancer, NSCLC)患者对EGFR-TK Is的治疗有良好反应，欧美地区NSCLC患者*EGFR*突变发生率约为10%-16%，亚裔NSCLC患者的*EGFR*突变发生率约为30%-50%。其突变主要发生在EGFR酪氨酸激酶结构域的ATP结合位点的编码区，即第18-21外显子，其中发生在19外显子的E746_A750缺失突变(属于15-bp/5AA缺失)和21外显子上的L858R点突变被称为经典型突变，约占*EGFR*突变的85%-90%^[13, 14]^。19外显子缺失突变包含如9-bp、12-bp、15-bp、18-bp、24-bp缺失突变等，其中以15-bp缺失的E746_A750del突变最为常见，约占19外显子缺失突变的68%，其余稍常见的少见型诸如18-bp缺失的L747_P753 > S(6.7%)，15-bp缺失的L747_T751del (6.4%)，9-bp缺失的L747_E749del(6.4%)等^[15]^。21外显子突变中，L858R点突变为最常见的类型，约占所有EGFR突变的39%^[16]^，其它少见型如L861Q突变等。除此之外，约有6%-10%为发生在18和20外显子的突变^[16]^。

2009年，Yu等[l7]在新西兰大白兔体内获得了两种单克隆抗体，即抗E746_A750缺失突变抗体和抗L858R点突变抗体，随后他们搜集了340例原发性NSCLC患者，并用这两种抗体测试上述肿瘤标本，所得的免疫组化结果与DNA直接测序结果比较显示灵敏度92%，特异度99%。近些年来，多项研究^[4, 15-24]^应用上述相同的两种抗体进一步检测NSCLC患者*EGFR*突变情况，免疫组化结果示灵敏度范围波动于24%-100%，特异度范围波动于77%-100%(表 1)。在表 1所列的11项有关免疫组化法检测特异性*EGFR*突变的研究中，均表现出较高的特异度，其中9项的特异度可高达96%及以上；但灵敏度浮动范围较大，最低仅有24%。回顾上述文献，分析影响免疫组化结果的原因，主要可能与免疫组化过程中抗原修复液的不同、免疫组化结果评判标准不同、是否加做总EGFR抗体检测等有关。下文将对上述主要影响因素做详细阐述。

**1:** 免疫组化法检测*EGFR*突变灵敏度及特异度的文献回顾（"/"表示相关数据缺少） Sensitivity and specificity of immunohistochemical detections of EGFR mutations in eleven studies ("/" indicates that data are not available)

References	*n*	E746-A750del			L858R			Overall	
		Sensitivity	Specificity		Sensitivity	Specificity		Sensitivity	Specificity
Jiang GY *et al*^[14]^	399	80%	85%		76%	92%		78%	87%
Brevet M *et al*^[15]^	194	67%	100%		76%	100%		/	/
Kato Y *et al*^[16]^	70	81%	100%		75%	97%		/	/
Yu J *et al*^[17]^	340	/	/		/	/		92%	99%
Simonetti S *et al*^[18]^	78	69%	100%		93%	100%		80%	100%
Kitamura A *et al*^[19]^	343	40%	99%		36%	97%		47%	96%
Wu SG *et al*^[20]^	143	94%	95%		88%	77%		/	/
Kozu Y *et al*^[21]^	577	42%	99%		76%	98%		/	/
Hofman P *et al*^[22]^	154	55%	97%		24%	98%		/	/
Ambrosini-Spaltro A *et al*^[21]^	33	67%	100%		100%	100%		61%	100%
Xiong Y *et al*^[24]^	50	59%	100%		81%	97%		/	/

## 1 抗原修复液的不同对免疫组化染色结果的影响

抗原修复是免疫组化染色中的重要步骤之一，通常用于福尔马林固定的石蜡包埋组织切片，由于组织中的部分抗原在甲醛固定过程中发生了蛋白之间的交联，加之醛基的封闭作用，从而使抗原决定簇暴露不完善。此时则需抗原修复液，使抗原决定簇充分暴露，以利抗原抗体结合，提高检测的阳性率^[25]^。

北京大学第一医院的熊焰等^[24]^在2013年研究免疫组化法检测*EGFR*突变的实验中，选取了三种不同的抗原修复液：柠檬酸钠溶液(pH 6.0)、EDTA(pH 8.0)和EDTA(pH 9.0)，分别用于50例经福尔马林固定、石蜡包埋的肺腺癌组织切片免疫组化染色过程中。染色结果示：EDTA(pH 8.0)处理过的组织切片显色最佳，即特异性染色强，背景色最浅；柠檬酸钠(pH 6.0)组阳性细胞染色太浅以致很难将其识别出；EDTA(pH 9.0)组背景染色太强以致难以区分出肿瘤细胞染色。读片病理医师间的一致度在EDTA (pH 8.0)组最高，柠檬酸钠(pH 6.0)组次之，EDTA(pH 9.0)组最低，并且其差异有统计学意义。究其理论依据：抗原在不同pH值环境中，等电点会发生改变，抗原抗体表面电荷的改变影响二者结合，造成在不同pH值的抗原修复液中染色强度不同^[25]^。

## 2 免疫组化染色结果的不同评判标准

目前对于免疫组化法的特异性染色结果尚无统一的评判标准，由于评分方法纷繁众多，尚缺乏大宗实验验证所有方法中哪种方法最优，但大部分方法都是以阳性细胞百分比与染色强度相结合的方式评判^[4, 15-24]^。阳性细胞标准有仅基于胞膜染色的^[15, 19, 23]^，也有基于胞膜和/或胞浆染色的^[4, 17, 18, 22]^。应用较多的是Yu等^[17]^，在大白兔体内获得两种特异性抗体之后，行特异性免疫组化染色所用标准。其将结果分为0-3+四个等级，以肿瘤细胞的胞膜和/或胞浆染色为基准，0为肿瘤细胞无染色或 < 10%的肿瘤细胞浅染；1+为 > 10%的肿瘤细胞浅染；2+为肿瘤细胞中度染色；3+为肿瘤细胞强染。1+-3+为阳性，0为阴性^[17]^。此后，Simonettis^[18]^、Hofman等^[22]^也应用同样方法判读结果，与分子水平的检测手段相比，均表现出较高的特异度，但灵敏度变化范围较大，低至24%，高至93%，考虑其差异还可能来源于评判标准以外的因素：比如所选样本中突变样本所占比例不同(所含突变样本越多，被检出突变的概率越高)；除最常见的19外显子的E746_A750缺失突变和21外显子的L858R点突变外，其它少见型突变由于不能与上述两种特异性抗体反应进而不能被检出。

2013年，熊等^[23]^针对特异性抗体检测EGFR突变的免疫组化染色，以DNA直接测序法为金标准，比较了三种评判标准各自的可靠程度。A法^[26]^以 > 10%的肿瘤细胞胞膜和/或胞浆中到强度染色为阳性；B法^[19]^以> 10%的肿瘤细胞胞膜任意强度染色为阳性；C法^[21]^以> 50%的肿瘤细胞胞膜和/或胞浆任意强度染色染色为阳性。结果示A法最为理想，应用此法可检测出E746_A750缺失突变和L858R点突变的特异度分别为100%和97%，灵敏度分别为59%和81%。从可靠程度来讲，对于L858R突变的检测，A法较B、C法有统计学差异；但对E746_A750缺失突变的检测，A法较B、C法无统计学差异^[23]^。总结A、B、C法，我们可以推出强调肿瘤细胞胞膜和/或胞浆染色的方法(A法)优于仅强调肿瘤细胞胞膜染色的方法(B法)，同时也优于仅考虑染色区域而忽略染色强度的方法(C法)。除此之外，还有将染色强度与阳性细胞百分比相乘的方法，以结果大于某一界定数值为阳性，小于该数值为阴性，如Colorado大学的免疫组化H评分法^[16]^和Kozu等^[21]^所使用的方法。

纵观上述文献，考虑免疫组化法检测*EGFR*特异性突变的特异度高，则误诊率较低；但灵敏度浮动范围较大，最低仅有24%，则提示其假阴性率较高，会有较多实际为*EGFR*突变的病例漏诊，此时则需借助其他灵敏度较高的分子水平手段进一步检测已明确是否存在*EGFR*突变。2013年，蒋等^[4]^针对免疫组化法检测*EGFR*特异性突变结果的特点，制定了一套*EGFR*突变筛查流程图。其收集了399例NSCLC患者的标本(包括145例手术标本，220例活检小标本和34例细胞学标本)，应用特异性抗体对上述标本进行免疫组化染色(对于活检小标本或细胞学标本，仅当肿瘤细胞数超过5个时才评价其免疫组化染色结果)，并将染色结果分为0-3+四个等级，标准为：0：无染色；1+：肿瘤细胞浅黄染不伴明显颗粒或不超过10%的肿瘤细胞黄染伴明显颗粒；2+：超过10%的肿瘤细胞黄染伴明显颗粒或不超过10%的肿瘤细胞棕染伴明显颗粒；3+：超过10%的肿瘤细胞棕染伴明显颗粒。另外应用TaqMan PCR法(较DNA直接测序灵敏度更高)作为金标准，检测每份标本各自EGFR突变情况。当以0和1+为阴性，2+和3+为阳性时，免疫组化法和TaqMan PCR法结果的一致度是最高的(*κ*=0.644)，然而在1+的评分里仍会有24例(24/104, 23.08%)为假阴性病例，在2+的评分里仍有33例(33/103, 32.04%)为假阳性病例，致免疫组化法的灵敏度为77.63%，特异度为86.64%，因此简单用此结果指导临床是不理想的。但在免疫组化染色评分为3+的标本中，特异度和阳性预测值均可达100%；免疫组化染色为评分为0的标本中，阴性预测值可达93.06%，加做总EGFR抗体(D38B1，非突变特异性抗体，可检测出所有EGFR蛋白表达无论其突变与否)检测，阴性预测值可高达97.22%。蒋等^[4]^根据上述结果的特点，制定的EGFR突变筛查流程为：若IHC染色结果评分为3+，可不行其他分子水平的检测，直接接受EGFR-TKI治疗；若评分为0，加做总EGFR抗体的免疫组化染色，筛查出的结果可达97%的阴性预测；当评分为1+或2+时，由于结果不可靠，需进一步接受分子水平的检测手段以明确是否存在*EGFR*突变。相比Yu等^[17]^对2+(中度染色)及3+(强染)级别仅设定笼统强度指标的方法，蒋等^[4]^对这两个级别进行了更为具体的百分比划分，因此考虑后者在指导病理医师对染色结果评分方面更为客观、严谨。另外蒋等^[4]^对不同类型的标本进行分析，推断免疫组化法用于手术切除标本优于活检小标本，活检小标本优于体液来源的细胞学标本。

目前大多数实验研究认为基于肿瘤细胞胞膜和/或胞浆的染色强度与染色区域的百分比设定的评分标准，并按程度分为0 -3+四个等级，是所有评分系统中最佳的方法^[4, 15, 17, 22, 24]^。尽管不同研究者实验所得免疫组化法检测突变灵敏度的结果波动范围较大且大多不甚理想，但这并不能成为限制其临床应用的障碍，根据蒋等^[4]^提出的流程图，当免疫组化法不能明确是否存在*EGFR*突变时，应进一步行灵敏度更高的分子水平检测手段验证。设置严格的免疫组化阳性标准可减少假阳性率，以避免患者因误诊予以TKI药物治疗而带来的损失。

## 3 加做EGFR单克隆抗体(D38B1)检测对免疫组化结果的影响

与突变特异性抗体不同的是，EGFR单克隆抗体(D38B1)可识别所有EGFR蛋白表达无论是否存在EGFR基因突变^[4]^。应用D38B1抗体检测出总EGFR表达为阴性的肿瘤标本，也不应被检测出特异性*EGFR*突变^[19]^。反之，特异性突变蛋白的表达水平受总EGFR表达水平影响，若肿瘤细胞内总EGFR表达水平很低，即便该样本的肿瘤细胞的确存在特异性*EGFR*基因突变，应用该特异性抗体行免疫组化染色的结果也可能为阴性，因此应用EGFR单克隆抗体(D38B1)检测总EGFR表达水平可减少特异性突变假阴性结果的产生，提高检测的灵敏度^[4, 19, 20]^。2011年Wu等^[20]^首次提出，加做总EGFR抗体(D38B1)检测并将其纳入对免疫组化结果的解释中可增加*EGFR*突变检测的可靠性，尤其对于21外显子的L858R点突变。其结论为，根据逻辑回归分析模型得出的最佳曲线下面积(area under the curve, AUC)，对于L858R的检测，综合L858R的Q评分(Q=强度×百分比)与总EGFR表达的Q评分得出的结果最佳，且与仅以L858R染色强度为评分标准的方法相比具有统计学差异(0.891 *vs* 0.853, *P*=0.036)。对于E746_A750缺失的检测，曲线下面积最优的方法为综合E746_A750缺失表达Q评分与总EGFR表达染色强度，但与仅以E746_A750表达强度为评分标准的方法相比，不具有统计学差异(0.969 *vs* 0.958, *P*=0.087)^[20]^。另外，Wu等^[20]^将免疫组化结果与患者临床服用靶向药物疗效相结合发现，上述免疫组化法阳性组的患者对EGFR-TKI治疗的反应及无进展生存期(progression free survival, PFS)均强于阴性组的患者。后来，蒋等^[4]^也发现，加做总EGFR抗体的免疫组化染色可以将E746_A750缺失突变和L858R点突变检出的灵敏度分别提高到82.56%和90%^[4]^。

## 4 其他

影响免疫组化结果可靠程度的因素有很多，然而其最终的可靠性取决于整个过程的质量控制，包括抗体的公司来源、免疫组化方法的选择、评分系统的选择、对结果的解释及是否能与分子水平检测方法的恰当结合等等^[2, 24]^。

## 5 小结

对于最常见的两种*EGFR*突变类型的检测，应用特异性抗体的免疫组化法具有快速、简便、经济、易于在众多病理实验室开展、可以保留形态学资料等特点，这些是其较目前新兴的分子水平检测手段最为明显的优势。另外，与经典的DNA直接测序相比，其更适于DNA含量较少的小标本检测，如在Kitamura^[19]^的实验中就曾发现一例*EGFR*基因检测为野生型，但免疫组化法结果阳性且对酪氨酸激酶抑制剂治疗反应良好的病例。因此不排除在那些与DNA直接测序法相比，免疫组化结果为"假阳性"的病例中，实际对酪氨酸激酶抑制剂治疗反应良好的病例，此时需借助灵敏度更高的分子水平检测进一步验证。合理应用特异性抗体的免疫组化法筛查*EGFR*突变具有实际临床意义，但要严格保证整个过程的质量控制，选择合理的试剂、制定恰当的结果评判标准、检测总EGFR表达水平等等。未来仍需大宗临床资料验证免疫组化法诊断阳性的患者，其靶向药物治疗反应。
